# Factors, influencing medication errors in prehospital care: A retrospective observational study

**DOI:** 10.1097/MD.0000000000018200

**Published:** 2019-12-10

**Authors:** Nikolai Ramadanov, Roman Klein, Urs Schumann, Abner Daniel Valdez Aguilar, Wilhelm Behringer

**Affiliations:** a Center for Emergency Medicine, University Hospital Jena, Friedrich Schiller; University Am Klinikum Jena; b Orthopedics, Trauma Surgery and Sports; Traumatology, Marienhaus Hospital Hetzelstift, Stiftstr. Neustadt; c Center for Internal Medicine, Clinic for Endocrinology and Diabetology Niemegker Str. Bad Belzig, Germany.

**Keywords:** emergency medical service, emergency medicine, prehospital emergency care, prehospital emergency physician

## Abstract

To determine the frequency of medication errors in prehospital care and to investigate the influencing factors – diagnostic agreement (DA), the medical educational status, the specialty, the approval for emergency medicine of the prehospital emergency physician, the patient age and sex and the time of deployment.

We retrospectively reviewed 708 patients from 2013 to 2015, treated by the prehospital emergency physicians of the emergency medical service center Bad Belzig, Germany. The medication appropriateness was determined by a systematic comparison of the administered medication in prehospital deployments with the discharge diagnosis, according to current guidelines. The influencing factors were examined by univariate analysis of medication appropriateness (MA), using the χ^2^, the Mann–Whtiney *U* and the Welch tests. We calculated a cut-off value with the Youden index to predict absent MA, according to patients age. The significance level was *P* = .05.

MA was absent in 220 of 708 patients (31.1%). In the case of present DA, MA was absent in 103 of 491 patients (20.9%). In the case of absent DA, MA was absent in 117 of 217 patients (53.9%) (*P* = .01). MA was absent in 82 of 227 patients (36.1%), treated by specialist and in 138 of 481 patients (28.7%), treated by resident physicians (*P* = .04). The calculated cut-off value to predict absent MA was 75.5 years. MA was absent in 100 of 375 patients (26.7%) of the younger patient age group (≤75.5 years), MA was absent 120 of 333 patients (36.0%) of the older patient age group (>75.5 years) (*P* = .01). Absent MA showed peak values (46.7%–60%) at night from 3 to 6 AM (*P* = .01) The other investigated factors had no influence on MA.

The correctness of medication as a quality feature in prehospital care shows a necessity for improvement with a proportion of 31.1% medication errors. The correct diagnosis by the prehospital emergency physician and his rapid accumulation of experience had an impact on the correctness of medication in prehospital care. Elderly patients (75+ years) and nighttime prehospital deployments (3–6 AM) were identified as high risk for medication errors by the emergency physicians.

## 1 Introduction

According to NCC MERP medication errors are “any preventable event that may cause or lead to inappropriate medication use or patient harm while the medication is in the control of the health-care professional, patient or consumer.”^[1]^ The German institution for medications and medicine products (BfArM) stated that preventable medication errors cause 500,000 admissions to the emergency department each year.^[2]^ Since medication errors often remain unreported we estimate even higher numbers.^[1]^

The following factors lead to medication errors: medication knowledge deficiencies 30%, patient knowledge deficiencies 29%, wrong calculations 18%, nomenclature issues 13%, other factors 10%.^[3]^ In addition, in prehospital care medication is often administered in urgent situations. This leads to three possible types of medication errors in prehospital care:^[1]^

### 1.1 Misuse

Inappropriate administration of medication due to incorrect doses, incorrect routes and contraindicated medications.

**Table 1:**
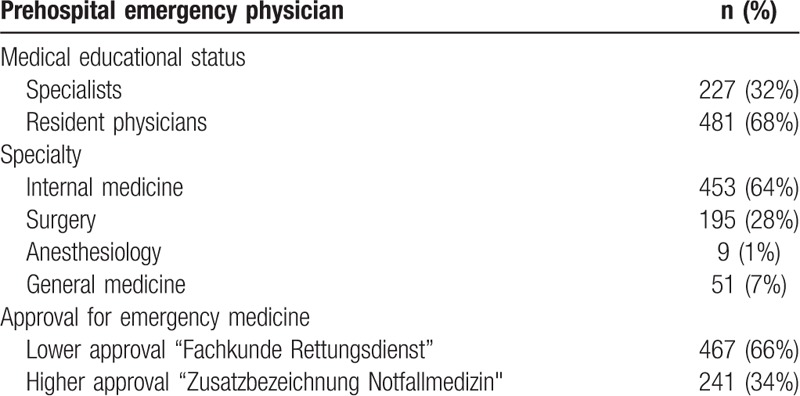
Listing of the deployment distribution according to medical educational status, specialty and approval for emergency medicine of the prehospital emergency physician, n = 708.

**Table 2:**
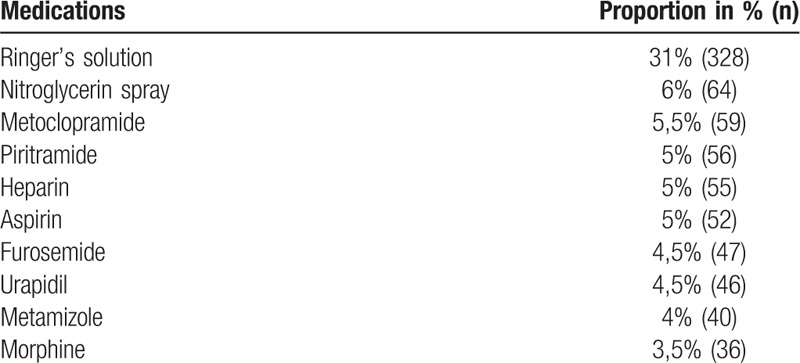
The 10 most common administered medications in prehospital care (n = 1058).

**Table 3:**
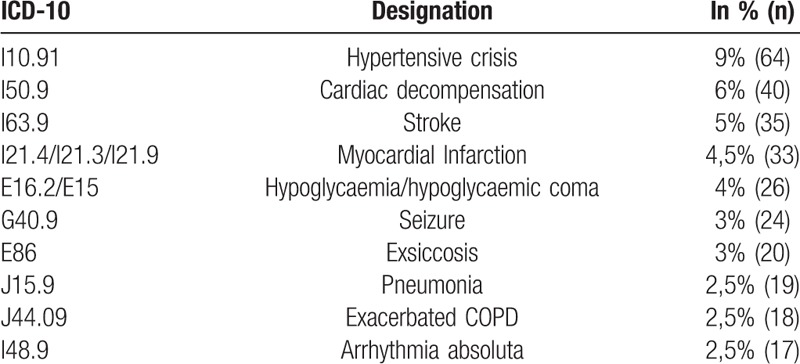
Ten most common deployment-related discharge diagnoses (n = 708).

### 1.2 Underuse

Omission of medication of proven benefit to the patient in the particular situation.

### 1.3 Overuse

Administration of unnecessary medication for the patient in the particular situation.

There are numerous studies on medication errors in general from all over the world.^[4–12]^ However, the literature on medication errors in prehospital care is insufficient. Since prehospital emergency medicine represents a very important area of medicine, there is a need for further research on this topic.

The aim of the present retrospective observational study was to determine the frequency of medication errors in prehospital care and to investigate the influencing factors.

## 2 Materials and methods

### 2.1 Description of the local EMS system

German emergency medical services (EMS) are an emergency physician led system. According to the rendez-vous principle, the prehospital emergency physician, accompanied by a paramedic with a vehicle on duty, meets the ambulance vehicle with 2 other paramedics at the scene. Prehospital emergency physicians are physicians with special education in emergency medicine. Emergency medicine is recognized as a supra-specialty to base medical specialties. There are 2 types of approval for emergency medicine: the older and lower approval “Fachkunde Rettungsdienst” and the current and higher approval “Zusatzbezeichnung Notfallmedizin”. Both give the physicians the permission to work in prehospital care. Furthermore, prehospital emergency physicians are physician of different specialty and educational medical status. Prehospital emergency physicians respond to a selected cohort of critical patients. Paramedics respond to the rest of all emergency calls. The deployment frequency at the EMS Center in Bad Belzig was comparatively low at 2.7 patients per day. The shift duration of the prehospital emergency physicians was 24 hours, starting at 7:30 AM At the EMS Center in Bad Belzig resident phyisicans had a clearly higher prehospital deployment frequency than specialists.

### 2.2 Data collection

The study investigated all prehospital deployments by emergency physicians at the EMS Center Bad Belzig from 2013 to 2015. Thus, all patient care reports of the EMS Center in Bad Belzig with the corresponding discharge summaries from the hospital Bad Belzig as well as from neighboring hospitals (Klinikum Ernst von Bergmann Potsdam, Asklepios Fachklinik Brandenburg, Städtisches Klinikum Brandenburg, Johanniter Krankenhaus im Fläming Treuenbrietzen) were collected. Patients or family members provided written informed consent and the Ethical committee of the University of Jena approved the data collection.

### 2.3 Exclusion of patients

**Figure 1:**
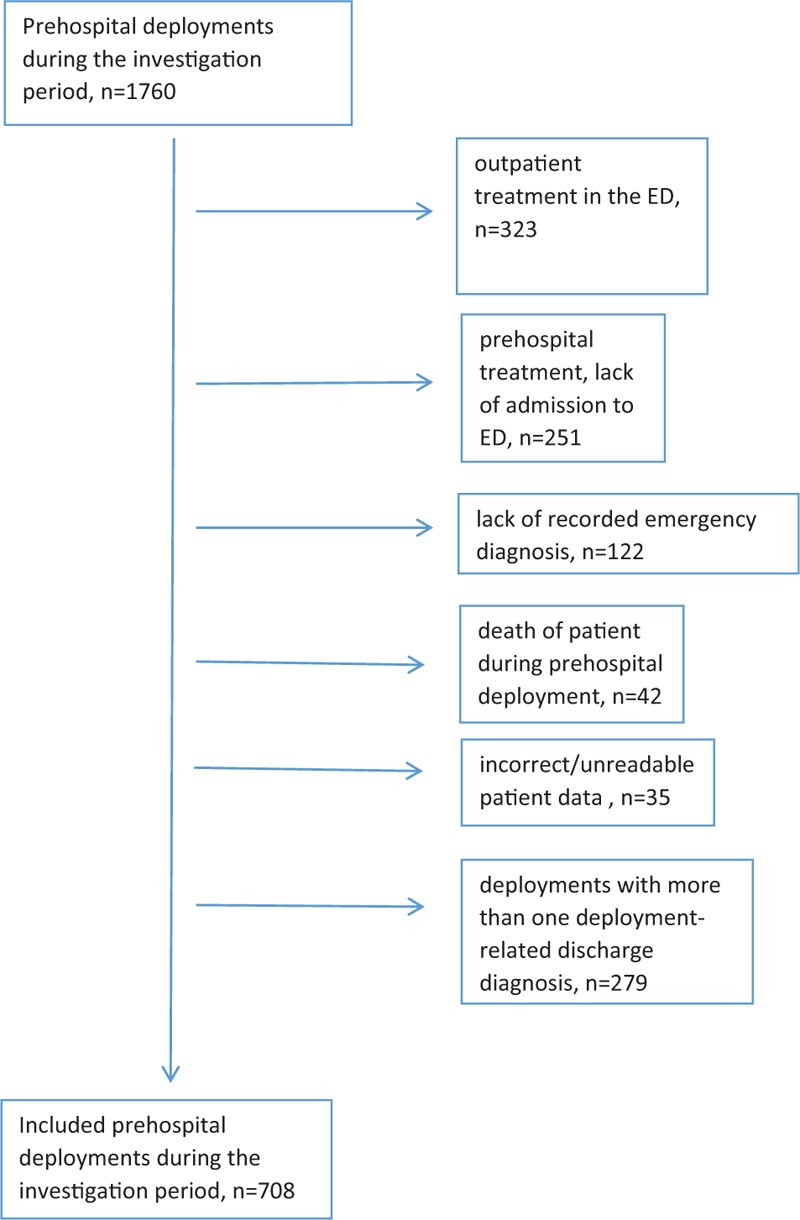
Inclusion chart.

First, all prehospital deployments with multiple deployment-related hospital discharge diagnoses were excluded, since otherwise the clear assignment of the prehospital medication to the corresponding diagnosis was impossible. Furthermore, patient care reports were excluded from the study for the following reasons: ambulant treatment in the emergency department (ED); prehospital treatment, lack of admission to the ED; lack of recorded emergency diagnosis; death of the patient during the deployment or incorrect/unreadable patient data. (Fig. 1)

### 2.4 Determination of medication appropriateness

Through a systematic comparison of prehospital medication to hospital discharge diagnosis, MA was determined. The determination of MA was carried out by the consensus of three experienced prehospital emergency physicians. There was an inter-rater reliability for MA of 0.96. In divergent cases, the judgment of the third emergency physician was consulted. MA was present when all the medications mentioned in the guidelines of the corresponding clinical picture were administered. MA was absent in case of omission of obligatory medication or administration of contraindicated medication according to current guidelines. The correctness of the dosage was not considered.

### 2.5 Influencing factors

The following factors were selected from routine data in order to investigate the influence on MA as extensively and objectively as possible:

1. Prehospital emergency physician related factors:
  - diagnostic agreement (DA)
  - medical educational status
  - specialty
  - approval for emergency medicine
2. Patient related factors
  - sex
  - age
3. Deployment related factors
  - time of deployment

### 2.6 Determination of diagnostic agreement

Through a systematic comparison of prehospital diagnosis to hospital discharge diagnosis, DA was determined after careful consideration of the entire course of each case from the emergency call to the completion of hospital treatment. The determination of DA was carried out by the consensus of the same prehospital emergency physicians, using the ICD 10 coding. There was an inter-rater reliability for DA of 1.0. Only deployment-related hospital discharge diagnoses were considered, that is, complications that occurred during the hospital stay of the patient were ignored as diagnoses in the discharge summaries.

### 2.7 Time of deployment

Time of deployment refers to the time of arrival at the scene. It was recorded rounded up to an entire hour.

### 2.8 Statistics

#### 2.8.1 Univariate analysis

The categorial variables for DA, the medical educational status, the specialty, the approval for emergency medicine of the prehospital emergency physician and the patient age were examined by univariate analysis of the categorial variable MA. For this purpose, the χ^2^ test was calculated. The non-normally distributed variables for patient age and time of deployment were examined by univariate analysis of the categorial variable MA, using the Mann-Whitney *U* Test. In addition, the patient age was tested with the Welch test. Using the Youden index, we calculated a cut-off value to predict absent MA. The following formula was used: Youden J = Sensitivity + Specificity − 1. According to the cut-off value, the patient age was divided into an older and a younger patient age group. Those 2 groups were examined by univariate analysis of the categorial variable MA, using the χ^2^ test. The significance level was *P* = .05.

#### 2.8.2 Multivariate analysis

Multivariate logistic regression analysis revealed weak models (Nagelkerke R^2^ = 0.02). The multivariate model had no explanatory value. All statistical calculations were performed using IBM SPSS Statistics 19 for Windows (IBM Germany GmbH, Ehningen).

## 3 Results

### 3.1 Patients

Overall, 337 of 708 (47.6%) patients were male and 371 of 708 (52.4%) patients female. The mean age of the patients was 68 (standard deviation ± 20) years (range: < 1 to 97 years). Overall, 220 of 708 (31.1%) patients took ≤4 medications per day on a regular basis and 488 of 708 (68.9%) patients took more than 4 different medications per day on a regular basis.

### 3.2 Prehospital emergency physicians

Table 1 shows the distribution of the deployments according to medical educational status, specialty and approval for emergency medicine of prehospital emergency physicians.

### 3.3 Description of the medication

Table 2 shows the 10 most commonly administered medications in the 708 patients included. In total, 1058 doses of medication and 37 different medications were administered by the prehospital emergency physicians.

### 3.4 Spectrum of prehospital deployment-related discharge diagnoses

Due to the upper mentioned exclusion criteria the study did not include patients with more than one prehospital deployment-related discharge diagnosis. Table 3 shows the 10 most common deployment-related discharge diagnoses.

### 3.5 Medication appropriateness

MA was absent in 220 of 708 patients (31.1%).

### 3.6 Factors, influencing the medication appropriateness

#### 3.6.1 Diagnostic agreement

DA was absent in 217 of 708 patients (30.6%). In the case of present DA, MA was absent in 103 of 491 patients (20.9%). In the case of absent DA, MA was absent in 117 of 217 patients (53.9%) (*P* = .01).

#### 3.6.2 Medical educational status

**Figure 2:**
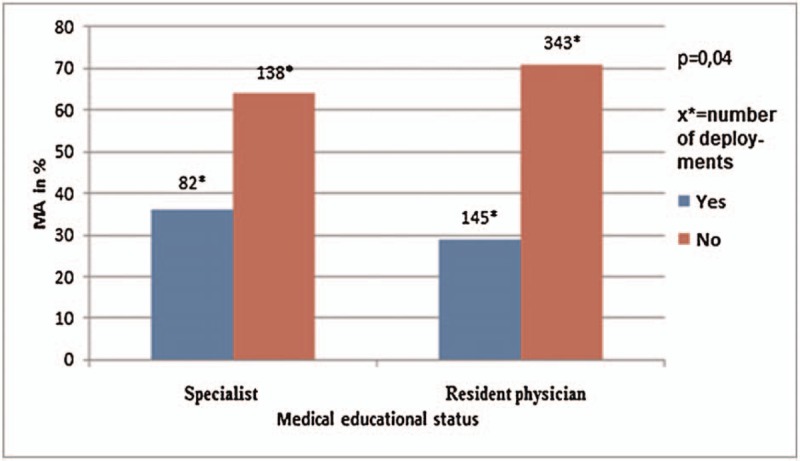
Representation of MA taking into account the medical educational status of the prehospital emergency physician.

Figure 2 shows the distribution of MA between emergency medication and hospital discharge diagnosis according to medical educational status. MA was absent in 82 of 227 patients (36.1%), treated by specialist and in 138 of 481 patients (28.7%), treated by resident physicians (*P* = .04).

#### 3.6.3 Patient age

**Figure 4:**
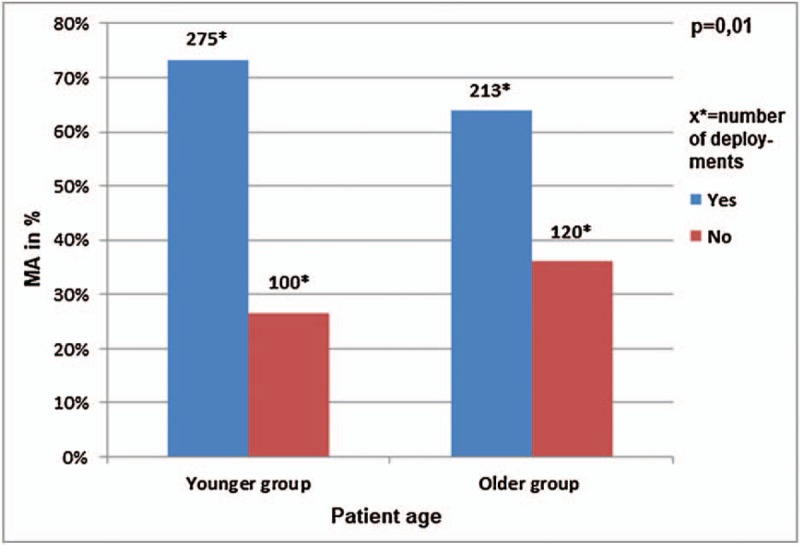
Representation of MA taking into account the patient age, divided in a younger and an older patient age group (cut-off value 75.5 years).

The mean age was 71 years (standard deviation ± 17) for patients with absent MA and 67 years (standard deviation ± 19) for patient with MA (Mann–Whitney *U* test *P* = .01 and Welch test *P* = .01). The calculated cut-off value to predict absent MA was 75.5 years. This is shown in Figure 4, where MA was absent in 100 of 375 patients (26.7%) of the younger patient age group (≤75.5 years) and MA was absent 120 of 333 patients (36.0%) of the older patient age group (>75.5 years) (*P* = .01).

#### 3.6.4 Time of deployment

**Figure 3:**
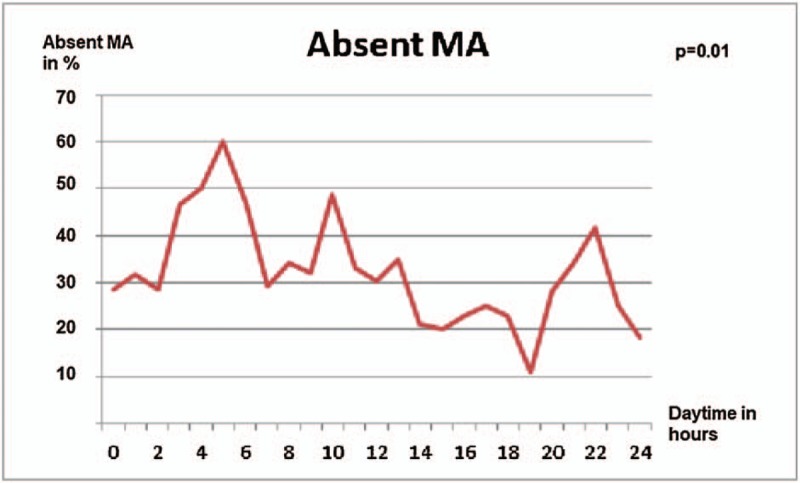
Fluctuation of absent MA over 24 hours in %.

Figure 3 shows the absent MA over the course of the day with peak values (46.7%–60%) at night from 3 to 6 AM (*P* = .01)

#### 3.6.5 Other factors

The specialty (*P* = .12) and the approval for emergency medicine of the prehospital emergency physician (*P* = .12) and the sex of the patient (*P* = .49) did not show any influence on MA.

## 4 Discussion

The correctness of the administered medication was estimated retrospectively in the present study through the calculation of MA. Our investigation showed, based on 708 included prehospital deployments, that the administered medication was incorrect in 31.1% of patients. In the literature, the results on medication discrepancies and medication errors show a very wide range from 9.1% to 77.5%.^[9–13]^ Since prehospital deployments often are a lifesaving task, a proportion of 31.1% medication errors must be viewed very critical. As possible measures to avoid medication errors, some of which are applicable in prehospital care, the following can be considered: strengthening the awareness of the patient and physician, improving communication between patient and physician, sufficient specialized information for the physician, involvement of pharmacists in the visit, consideration of the pre-existing conditions and premedication of the patient as well as promotion of a safety culture.^[14,15]^ The rule of “the six rights” is a simplified aid to minimize medication errors:^[1]^

1. Right medication
2. Right dose
3. Right route
4. Right time
5. Right patient
6. Right documentation

We were able to identify 4 of 7 investigated factors with influence on medication errors. There was a significant influence of the diagnosis by the prehospital emergency physician on medication errors in prehospital care. This is understandable, since in cases of incorrect diagnosis, the correct medication can be randomly administered to the particular patient.

Further, there was a significant influence of the medical educational status of prehospital emergency physicians on medication errors in prehospital care. Resident physicians with 28.7% medication errors achieved better results than specialists with 36.1% medication errors. We explain the results with the higher prehospital deployment frequency of residents. The important role of the rapid accumulation of experience in younger prehospital emergency physicians was already noticed in other studies.^[16,17]^

There was a significant influence of patient age on medication errors in prehospital care. We calculated a cut-off value for prediction of medication errors at the age of 75 years. Medication errors occurred in 26.7% of the younger patient age group (≤75 years) and in 36.0% of the older patient age group (76+ years). Our study identified older patients as a group of high risk patients for medication errors by prehospital emergency physicians. Medication related problems, medication discrepancies and errors in elderly patients are known in literature.^[4,5,21–24]^ They lead to higher morbidity, mortality and economic impact.^[21]^ Polypharmacy, age-related pharmacokinetic and pharmacodynamic changes are some of the reasons that place elderly patients at a higher risk.^[21]^

Our findings showed clear fluctuation of medication errors over 24 hours. Peak values of medication errors were reached in a nonphysiological working time at night from 3 to 6 AM The shortage of sleep and the lack of recovery after a full day on duty might be considered as reasons. A study of 580 patients on diagnostic agreement in prehospital care came to comparable results. The incorrect diagnosis by the prehospital emergency physician showed a similar fluctuation over 24 hours with peak values at 4 and 5 AM.^[18]^ Another study in a pediatric ward showed a significant increase in medication error rate by nurses during evening and nighttime shifts in comparison to the rest of daytime.^[19]^ A systematic review analyzed recent literature regarding nurse night shift errors. They found an increase of multiple kind of nurse errors at night and gave several possible causes and solutions. Some of them are applicable to prehospital emergency physicians as well.^[20]^

We identified some limitations of this study. A generalization of the results is limited due to the inhomogeneous emergency medical structure at the EMS centers over the German federal states. The informative value of the specialization for anesthesiologists and general practitioners is limited due to their low proportion of the total number of prehospital deployments.

## 5 Conclusions

The correctness of medication as a quality feature in prehospital care shows a necessity for improvement with a proportion of 31.1% medication errors. The correct diagnosis by the prehospital emergency physician and his rapid accumulation of experience had an impact on the correctness of medication in prehospital care. Elderly patients (75+ years) and nighttime prehospital deployments (3–6 AM) were identified as high risk for medication errors by the emergency physicians.

## Author contributions

**Conceptualization:** Nikolai Ramadanov, Wilhelm Behringer.

**Data curation:** Nikolai Ramadanov.

**Formal analysis:** Nikolai Ramadanov.

**Funding acquisition:** Nikolai Ramadanov.

**Investigation:** Nikolai Ramadanov.

**Methodology:** Nikolai Ramadanov, Wilhelm Behringer.

**Project administration:** Nikolai Ramadanov.

**Resources:** Nikolai Ramadanov.

**Software:** Nikolai Ramadanov.

**Visualization:** Nikolai Ramadanov, Urs Schumann, Abner Daniel Aguilar Valdez.

**Writing – original draft:** Nikolai Ramadanov, Roman Klein, Wilhelm Behringer.

Nikolai Ramadanov orcid: 0000-0003-4669-8187.
